# The sympathetic nervous system shapes the tumor microenvironment to impair chemotherapy response

**DOI:** 10.3389/fonc.2024.1460493

**Published:** 2024-09-24

**Authors:** Annabel V. Manoleras, Erica K. Sloan, Aeson Chang

**Affiliations:** Drug Discovery Biology, Monash Institute of Pharmaceutical Sciences, Monash University, Parkville, VIC, Australia

**Keywords:** cancer, sympathetic nervous system, chemotherapy, beta-blocker, metastasis, neuron

## Abstract

The tumor microenvironment influences cancer progression and response to treatments, which ultimately impacts the survival of patients with cancer. The sympathetic nervous system (SNS) is a core component of solid tumors that arise in the body. In addition to influencing cancer progression, a role for the SNS in the effectiveness of cancer treatments is beginning to emerge. This review explores evidence that the SNS impairs chemotherapy efficacy. We review findings of studies that evaluated the impact of neural ablation on chemotherapy outcomes and discuss plausible mechanisms for the impact of neural signaling on chemotherapy efficacy. We then discuss implications for clinical practice, including opportunities to block neural signaling to improve response to chemotherapy.

## Introduction

The tumor microenvironment was long thought to be a bystander in cancer progression but is now recognized to play an active role in regulating cancer progression (1). Cancer cells interact with stromal and immune cells in the tumor microenvironment, actively sculpting the environment to favor cancer progression (1). Multiple compartments of the tumor microenvironment regulate both treatment efficacy and resistance (2, 3). For example, variation in the immune profile of individual tumors predict treatment outcomes, where high T cell and low macrophage abundance in the tumor has been linked to better response to chemotherapy in patients (4, 5). Moreover, vascular integrity within the tumor can affect chemotherapy distribution within the tumor (6, 7). Whilst much is known about how the vasculature and immune landscape of tumors contributes to treatment response or resistance, the role of tumor-associated nerves in modulating these effects is only beginning to be elucidated.

The peripheral nervous system is comprised of the autonomic (sympathetic and parasympathetic) and somatic nervous systems. The sympathetic and parasympathetic nervous systems regulates physiological functions of organs, while sensory neurons transmit internal and external sensory cues (temperature, touch, mechanical and noxious stimuli) to the brain, and regulate signaling in innervated organs through neuropeptide release (8, 9). Tumors that arise in the body (outside the central nervous system) may be innervated by more than one neural subtype (10–15), each of which has been shown to play an active role in cancer progression by either regulating cancer cell function or shaping the tumor microenvironment to favor cancer progression, as reviewed previously (16).

In addition to modulating cancer progression, recent studies provide evidence that the sympathetic nervous system (SNS) also regulates the response to chemotherapy treatment. The SNS is activated by stimuli that challenge organismal homeostasis, inducing a fight-or-flight stress response (17). Activation of the SNS induces the release of catecholaminergic neurotransmitters including norepinephrine (or noradrenaline) from nerve endings and epinephrine (or adrenaline) from the adrenal gland (18). Neurotransmitter ligation of G-protein coupled adrenergic receptors shifts gene expression to modulate the behavior of diverse cell types in the tumor microenvironment. The β_2_-adrenergic receptor (β_2_AR) subtype is expressed by tumor cells, endothelial cells, and immune cells, and preclinical studies from the last two decades show that β_2_AR signaling drives tumor growth and metastasis and suppresses anti-cancer immunity in various cancer types (19–33), as previously reviewed (34). More recently, evidence for the role of other adrenergic receptor subtypes (α_1_AR, α_2_AR, β_1_AR and β_3_AR) in regulating cancer progression is emerging (35–38). As described below, evidence for SNS regulation of chemotherapy response has focused on the role of β_2_AR, and the role of the other adrenergic receptors on chemotherapy response is yet to be explored. Therefore, here we focus on evidence that SNS signaling through β_2_AR modulates chemotherapy response.

## Targeting tumor-associated nerves improves response to chemotherapy

Expanding on well characterized roles for the SNS in cancer progression (16, 39), the application of tools from neuroscience and pharmacology to the field of cancer biology has begun to elucidate a role for the SNS in response to cancer treatments including chemotherapy. Evidence that SNS signaling can impact the effectiveness of chemotherapy comes from studies that target different aspects of SNS signaling including tumor-associated nerves, neurotrophins that support these nerves, and the receptor signaling activated by SNS neurotransmitters.

**Figure 1:**
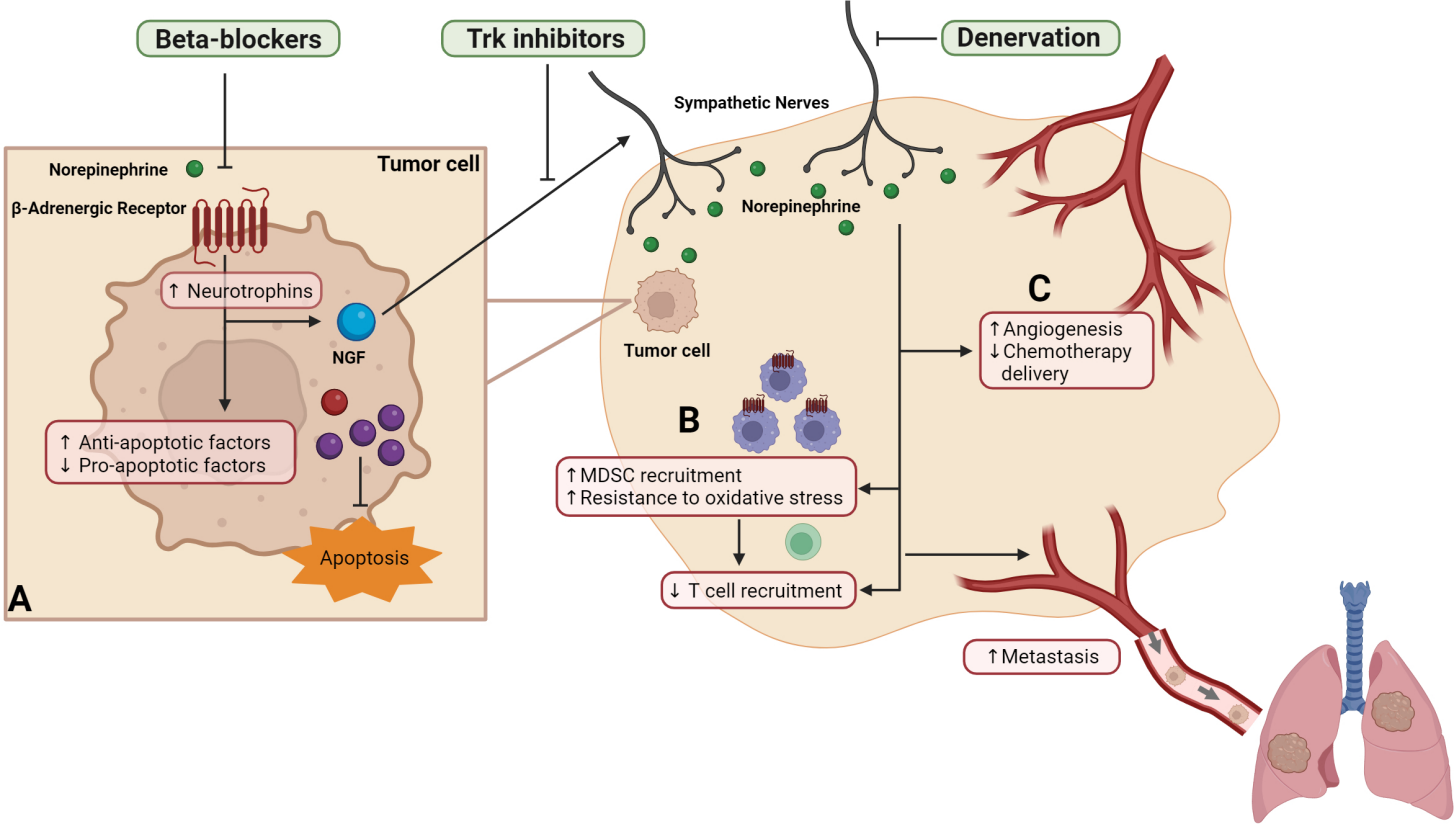
Strategies that block SNS signaling during chemotherapy improve cancer-related outcomes. Sympathetic signaling acts on the multiple components of the tumor microenvironment to drive cancer cell dissemination, including **(A)** tumor cells to increase transcription of neurotrophins including nerve growth factor (NGF) and shift the balance of pro- and anti-apoptotic factors to reduce apoptosis, **(B)** immune cells to increase recruitment of myeloid derived suppressor cells (MDSC) to the tumor, increase MDSC resistance to oxidative stress, and reduced cytotoxic T cells recruitment, and **(C)** blood and lymph vasculature to reduce drug delivery and increase pathways of tumor cell escape. Preclinical studies showed that interventions that target sympathetic nervous system, including surgical or chemical denervation, Trk inhibitors or beta-blockers reduce metastasis and improve survival.

In mouse models of triple-negative breast cancer (TNBC) and pancreatic ductal adenocarcinoma, localized SNS denervation of the tumor microenvironment using surgery or neurotoxin prior to chemotherapy treatment were shown to improve chemotherapy control of metastasis and increase overall survival (23, 28) (**Table 1**). In a mouse model of TNBC, ablation of sympathetic nerves within the mammary fatpad using 6-hydroxydopamine reduced metastasis progression after treatment with the anthracycline chemotherapy doxorubicin (23) (**Figure 1**). In contrast, anthracycline chemotherapy had no effect on metastasis in mice with intact sympathetic innervation at the site of the primary tumor. Denervation of the primary tumor had no effect on doxorubicin control of primary tumor growth, nor did denervation of the primary tumor have any effect on metastasis in mice that were not treated with chemotherapy. These findings indicate that neural signaling may modulate the response to chemotherapy by affecting the invasive properties of tumor cells rather than by enhancing the effects of chemotherapy on tumor cell killing (23). Similarly, resection of the nerve bundles that supply the pancreas (around the celiac and superior mesenteric arteries) in mice with established pancreas tumors improved the response to subsequent treatment with gemcitabine by doubling overall survival compared to mice with intact nerves (28). As the effect of nerve resection on survival was not evaluated in mice not treated with gemcitabine, it is possible that improved survival was due to an additive effect of chemotherapy and denervation, rather than an effect of neural ablation on gemcitabine efficacy. Additionally, this study did not confirm that the resected nerve bundle specifically contained sympathetic nerve fibers, hence ablation of other neuronal subtypes present in the nerve bundles may contribute to the observed improvement in survival outcomes (28). Nonetheless, these studies suggest that neural signaling may impair the effect of chemotherapy.

**Table 1:** Neural-targeted interventions modulate cancer progression and response to chemotherapy in mouse models of cancer.

Cancer	Neural intervention (vs. control)	Chemotherapy (vs. control)	Neural intervention + chemotherapy (vs. chemotherapy alone)	Ref
Receptor signalling intervention				
TNBC	**Propranolol** (non-selective βAR antagonist) Tumor: no effect Metastasis: no effect	**Doxorubicin** Tumor: ↓ Metastasis: no effect	**Propranolol + Doxorubicin** Tumor: no effect Metastasis: ↓	23
	***ADRB2*-deficient tumor cells** Tumor: no effect Metastasis: no effect	**Doxorubicin** Tumor: ↓ Metastasis: no effect	***ADRB2*-deficient tumor cells + doxorubicin** Tumor: no effect Metastasis: ↓	
PDAC	**ICI-118551 β_2_AR antagonist** Not assessed	**Gemcitabine** Not assessed	**ICI118551 + Gemcitabine** Improved survival Tumor: ↓	28
Denervation				
TNBC	**6-OHDA** Tumor: no effect Metastasis: no effect	**Doxorubicin** Tumor: ↓ Metastasis: no effect	**6-OHDA + Doxorubicin** Tumor: no effect Metastasis: ↓	23
PDAC	**Ganglionectomy** Not assessed	**Gemcitabine** Not assessed	**Ganglionectomy + Gemcitabine** Improved survival	28
Neurotrophin targeted intervention				
TNBC	**NGF-deficient tumor cells** Tumor: ↓ Metastasis: ↓	**Doxorubicin** Tumor: ↓ Metastasis: no effect	**NGF-deficient tumor cells + doxorubicin** Tumor: ↓ Metastasis: ↓	23
PDAC	**PLX-7486** (pan-Trk inhibitor) Not assessed	**Gemcitabine** Not assessed	**PLX-7486 + Gemcitabine** Improved survival	28
Colon cancer	**Pro-BDNF neutralizing antibody** Not assessed	**5-fluroruracil** Tumor: ↓	**Pro-BDNF neutralizing antibody + 5-fluroruracil** Tumor: ↓	40

Triple-negative breast cancer (TNBC): 4T1.2 or MDA-MB-231^HM^ orthotopic mammary tumor. Pancreatic Ductal Adenocarcinoma (PDAC): KPC transgenic. Colon: CT26 orthotopic tumor. 6-OHDA, 6-hydroxydopamine; BDNF, Brain-derived neurotropic factor. ↓: Decreased.

Studies that targeted neurotrophin support of tissue innervation also provide evidence that neural signaling impacts chemotherapy response. Neurotrophins including nerve growth factor (NGF) support the growth and maintenance of sympathetic neurons (40). Within tumors, neurotrophins are synthesized by cancer cells and could increase tumor innervation (41, 42). Blocking neurotrophin signaling using a pan-tropomyosin receptor kinase (Trk) inhibitor during treatment with gemcitabine was shown to improve survival of mice with pancreatic cancer compared with treatment with gemcitabine alone (28) (**Table 1**). Similarly, blocking pro-brain derived neurotrophic factor using a neutralizing antibody during treatment with 5-fluorouracil reduced growth associated protein-43-positive tumor innervation and resulted in smaller colorectal tumors than treatment with the chemotherapy alone (41) (**Table 1**). In addition to being expressed by neurons, TrkA and p75 neurotrophin receptors (p75^NTR^) are expressed by tumor cells and NGF activation of these receptors promotes tumor cell proliferation (28, 43). Therefore, it will be important to determine whether improved outcomes of neurotrophin targeted strategies that have been observed in preclinical studies are due to an effect on chemotherapy efficacy or a result of limiting cancer cell proliferation during treatment.

When activated, SNS nerves release neurotransmitters that bind to adrenergic receptors in target tissues. Evaluation of clinical cancer samples shows wide variation in levels of adrenergic receptors on tumor cells (44, 45). A number of studies that examined the prognostic potential of β_2_AR expression found that high tumor cell β_2_AR was associated with worse survival outcomes in various cancer types (32, 46–50). A plausible mechanism for this effect may be through modulation of treatment efficacy. For example, in a study of HER2^+^ breast cancer, high levels of tumor cell β_2_AR prior to neoadjuvant treatment with anthracycline-containing chemotherapy and trastuzumab was associated with decreased pathological complete response (46). While not all tumors express high β_2_AR at diagnosis, there is evidence that β_2_AR levels (and therefore sensitivity to SNS activation) may be increased by chemotherapy (23). Anthracycline drugs including doxorubicin and epirubicin were shown to increase β_2_AR transcription in mouse models of TNBC and in patient samples, thereby sensitizing tumor cells to SNS neurotransmitter signaling (23). These findings suggest that chemotherapy treatment could potentially affect the tumor response to future treatment by modulating sensitivity to neural signaling.

In line with these clinical observations, studies that investigated pharmacological blockade of βAR suggest that the SNS impacts chemotherapy outcomes. β-adrenergic antagonists, also known as beta-blockers, are commonly used to treat cardiovascular diseases and a range of other conditions (51). In mice with TNBC, treatment with the beta-blocker propranolol significantly improved doxorubicin-mediated control of metastatic growth, independent of an effect on the primary tumor (23) (**Table 1**). Genetic knockout of β_2_AR from tumor cells replicated the effect of beta-blockade: tumors derived from β_2_AR-deficient tumor cells had reduced metastasis following doxorubicin administration compared to tumors derived from β_2_AR-expressing tumor cells (23). Other chemotherapy drugs may also be improved by blocking neural signaling. In an immunocompromised mouse model of TNBC, propranolol-mediated blockade of SNS signaling during treatment with paclitaxel and 5-fluorouracil improved survival of mice (determined by primary tumor burden) (52), and treating mice with pancreatic tumor with the selective β_2_-blocker ICI-118551 during treatment with gemcitabine slowed primary tumor growth and increased the overall survival time compared treatment with gemcitabine chemotherapy alone (28) (**Table 1**).

Retrospective clinical studies that investigated cancer-related outcomes in patients who were treated with beta-blockers at the time of cancer diagnosis or treatment also support a role for βAR signaling in improving clinical outcomes. Beta-blockers are used to treat cardiovascular disease including arrhythmias and heart failure, glaucoma, and are used for migraine prophylaxis (53). As a consequence of the widespread use of beta-blocker drugs, a significant proportion of adults who are diagnosed with cancer will (co-incidentally) be prescribed a beta-blocker during cancer treatment. A recent retrospective study using both hospital and population cohorts, found that beta-blocker use at the time of treatment with chemotherapy was associated with better metastasis-free survival in women with TNBC compared to no use of beta-blockers (23). Similar findings were reported in another cohort of women with TNBC treated with chemotherapy (54). These studies did not distinguish between use of cardio-selective versus non-selective beta-blockers. While beta-blocker use at diagnosis has been associated with improved disease-free survival and/or overall survival in other cancer types (55–63), those studies did not consider interactions with standard cancer treatments such as chemotherapy. Nonetheless, as beta-blocker use for cardiovascular indications tends to be long-term, it is likely that beta-blockers were used concurrently with cancer treatment in those cancer cohorts. In future clinical analyses it will be important to investigate if the beneficial effects of beta-blockers on cancer outcomes is due to an interaction with chemotherapy.

## Mechanisms for neural effects on chemotherapy treatment

The effects of SNS activation on chemotherapy response may be due to effects on tumor cells, or components of the tumor microenvironment (**Figure 1**). While mechanisms of chemotherapy drugs vary, their ultimate goal is to induce apoptosis in cancer cells. In addition, optimal chemotherapeutic effects may be dependent on induction of a tumor-targeted immune response and drug access to the tumor. Neural signaling may impact the effects of chemotherapy by acting at each of these levels.

Beta-adrenergic signaling desensitizes cancer cells to the cytotoxic effects of chemotherapy, an effect that is prevented with beta-blockers (24, 52, 64–67). This has been demonstrated for different chemotherapy drugs including cisplatin, paclitaxel, and vincristine, and in cell lines derived from different cancer types including breast, ovarian, pancreatic cancer and neuroblastoma (24, 52, 64–67). Several mechanisms have been proposed, including modulation of the apoptotic molecular machinery and DNA damage response by βAR signaling (**Figure 1A**) (24, 63, 65, 67). Preclinical studies have shown that βAR signaling inactivated pro-apoptotic protein BAD and p53, and increased anti-apoptotic protein BCL-X_L_, BCL-2 and MCL1 in pancreatic cancer cells (24). βAR signaling can also increase dual specificity phosphatase 1 protein which inhibited paclitaxel-induced JNK/c-Jun-dependent apoptotic signaling pathway (64). βAR signaling has been implicated in promoting DNA damage, thus driving G1 cell cycle arrest in breast cancer cells which may contribute to the lack of paclitaxel efficacy in the presence of βAR signaling (66). In contrast, blockade of βAR signaling using propranolol increased levels of the pro-apoptotic protein p53 in breast cancer cells *in vitro*; and a similar observation was reported in a single patient who was treated with propranolol before surgical resection of tumor (68). These findings indicate that blockade of βAR signaling may improve cytotoxic effects of chemotherapy. Supporting this hypothesis, co-administration of propranolol increased tumor sensitivity to docetaxel or pro-apoptotic therapy Apo2L/TRAIL, resulting in smaller tumors compared to mice that were treated with either therapy alone (24, 64).

The finding that blocking sympathetic neural signaling improve chemotherapy control of metastasis without impacting primary tumor burden *in vivo* or cancer cell proliferation *in vitro* (23), suggests that beta-blockade may improve chemotherapy efficacy via alternative mechanisms besides targeting chemotherapy-induced apoptosis. Emerging evidence suggests that neural signaling can impair chemotherapy through effects on immune cells (69)(**Figure 1B**). An immune response is required for the optimal effect of some chemotherapy drugs (70, 71). For example, the anthracycline drug doxorubicin increased proliferation of tumor-specific CD8+ T cells in tumor-draining lymph nodes and T cell deletion reduced the effectiveness of chemotherapy in mice (71). Moreover, leukocyte infiltration into tumors predicts recurrence-free and overall cancer survival in TNBC patients who received adjuvant chemotherapy (5). However, myeloid-derived suppressor cells (MDSC) that are prevalent in the tumor can dampen CD8 immune response (72), which may affect chemotherapy efficacy. Recently, it was shown that activation of βAR signaling in MDSC inhibited doxorubicin-induced apoptosis by inducing metabolic reprogramming, thus making the MDSC more resistant to doxorubicin-induced oxidative stress (**Figure 1B**) (69). Consequently, propranolol blockade of βAR signaling promoted MDSC cell death and reduced MDSC abundance in tumors, which was associated with increased survival of mice with lymphoma compared to treatment with doxorubicin or propranolol alone (69). Neural signaling through βAR has also been shown to regulate recruitment, expansion and function of other immune cell populations. For example, βAR signaling promotes recruitment of other myeloid cell populations to tumors, while reducing recruitment of functional CD8+ T cells (29, 73–76). Additionally, βAR signaling can modulates T cell motility by regulating tissue oxygen levels, thus affecting T cell priming and expansion of tumor-specific T cells (19). Neural signaling also impairs T cell priming and thus cytotoxicity by downregulating the antigen presentation capacity of dendritic cells (20, 77). Notably, most of the immune cells regulated by neural signaling through βAR have been implicated in modulation of chemotherapy efficacy (4, 70, 71, 78–81). Therefore, future studies are needed to examine if these immune cells are also targeted by βAR signaling in the context of chemotherapy treatment. These studies will provide important insights into how blocking neural signaling can improve the anti-cancer immune response, which may have significant implications for clinical practice with the recent shift towards addition of immunotherapy to chemotherapy regimens.

Neural regulation of tumor vasculature may also impact the effect of chemotherapy (**Figure 1C**). The tumor vasculature serves as an important route to ensure effective drug delivery (6, 7). Physiological SNS activation by restraint stress has been shown to dysregulate blood and lymph vasculature within tumors via βAR-signaling (29, 30, 60, 82). βAR-induced remodeling of vasculature in tumor occurs through both direct effects on endothelial cells and indirect effects through immune cells and tumor cells (29, 30, 33, 60, 82). Genetic deletion of *Adrb2*, which encodes β_2_AR, in endothelial cells significantly increased oxidative metabolism in endothelial cells, thus reducing angiogenesis, vascular density, and ultimately progression of prostatic intraepithelial neoplasia in a transgenic model of prostate cancer (33). Other studies have shown that tumor-associated macrophages are required for βAR-induced vascular remodeling by inducing production of VEGF-A and VEGF-C by tumor cells (29, 60). βAR signaling can also affect tumor cells directly by upregulating transcription and production of VEGF-A, leading to vascular remodeling in tumors (30, 82, 83). As tumor vasculature is a critical component of the tumor microenvironment that impacts chemotherapy efficacy (80, 81, 84), βAR-induced remodeling of tumor vasculature may contribute to the effects of neural signaling on chemotherapy efficacy. Therefore, to fully understand how neural signaling modulates the effects of chemotherapy, it will be important for future studies to define how the SNS regulates multiple aspects of the tumor microenvironment including immune and vasculature contributions to treatment response.

## Discussion: clinical implications and future directions

SNS control of chemotherapy efficacy suggests that strategies that target neural signaling may be leveraged to improve outcomes for patients with cancer. Pharmacological beta-blockade could be rapidly translated to help patients as beta-blockers are widely available, inexpensive, and well-tolerated drugs that could be readily combined with existing treatments including chemotherapy. Early-stage clinical trials for the use of beta-blockers are promising. Phase II window of opportunity studies have shown that the beta-blocker propranolol reduces biomarkers of invasion and inflammation and is well tolerated by patients with cancer (85–87). Feasibility trials have demonstrated that propranolol may be safely combined with neoadjuvant chemotherapy (88, 89) and used in the perioperative period (85, 86). Additional insights may come from several ongoing single arm trials (NCT04005365, NCT02641314, NCT03108300, NCT02897986) in other cancer types, and early evidence suggests that beta-blockers may improve progression-free survival in cancer patients (90).

To best design future clinical trials for evaluation of beta-blocker use in combination with existing cancer therapies, we need to understand which treatment regimens can be optimally enhanced by blocking sympathetic neural signaling. To address this, a number of key questions remain to be addressed. Firstly, does the SNS broadly impair chemotherapy, or are effects limited to drugs with specific mechanisms of action? In the context of TNBC, observations from a hospital cohort and mechanistic preclinical studies suggest that beta-blockers may optimally improve anthracycline-containing regimens (23, 54). However, *in vitro* and *in vivo* studies across TNBC, neuroblastoma, pancreatic cancer, and osteosarcoma, suggest that beta-blockers may improve the effect of taxanes, 5-fluorouracil, vincristine, gemcitabine and cisplatin (28, 52, 91, 92). Therefore, it will be important to determine if the benefit of blocking neural signaling depends on the distinctive biology of different cancer types.

Second, consideration of the receptor that mediates the effects of SNS activation will determine the type of beta-blocker used in future clinical trials. The mechanistic studies described above emphasize a role for β_2_AR in the effects of neural signaling on chemotherapy (23, 69). This raises the possibility that non-selective beta-blockers may be more effective in the chemotherapy treatment context than β_1_AR-targeted cardio-selective beta-blockers. Beyond the treatment context, some epidemiological analyses support this concept (55, 57, 93), while other studies that did not distinguish between beta-blocker subtypes have found improved cancer-related outcomes (59, 62, 94). It will be also important to consider the timing of beta-blockade. Previous phase II trials were designed to block SNS signaling in the peri-operative period (85, 86), and emerging studies suggest that beta-blockade during neoadjuvant chemotherapy treatment may improve long term relapse-free and overall survival (23, 54). Recent findings that SNS signaling through α_2_AR may have anti-tumor effects by modulating the immune response, has led to suggestions that drugs such as clonidine may be effective in cancer (36). However, perioperative clonidine use during resection surgery for lung or breast cancer was found to have no association with cancer-related survival (95). Therefore, further research on the role of α_2_AR in chemotherapy response is needed. Third, does SNS activity affect other cancer treatment modalities, such as immunotherapy, radiotherapy, or targeted therapies? Preclinical studies suggest that this is likely, indicating that beta-blockade may have benefit across different cancer treatments. Physiological SNS activation by exposure to cold temperature housing impaired the therapeutic effects of immune checkpoint inhibitors in mouse models of breast cancer, melanoma and lymphoma, and this effect was phenocopied by βAR agonism and blocked by beta-blockade (20, 76, 96). Moreover, preclinical studies found that beta-blockade improved radiotherapy-induced reduction of primary tumor growth and enhanced an abscopal response in a non-irradiated second primary tumor, suggesting that βAR signaling impairs the efficacy of radiotherapy (97, 98). Other targeted therapies including trastuzumab, erlotinib, and the anti-angiogenic drug sunitinib are impaired by βAR signaling (46, 82, 99, 100). Mechanistic studies suggest that βAR activation induced oncogenic PI3K/Akt/mTOR signaling or LKB1/mTOR signaling, which impaired the response to trastuzumab and erlotinib, respectively (46, 100), while βAR upregulation of angiogenic factors VEGF, IL-8, and IL-6 may impair the effects of sunitinib (82, 99). Expanding our understanding of the impact of βAR signaling on other treatment modalities, beside chemotherapy, will help to guide strategic translation of neural-targeted intervention strategies including beta-blockers.

Beyond evaluation of the SNS, an understanding of the role of other peripheral neural subtypes may provide additional opportunities to enhance cancer treatment response. A growing body of work shows that sensory neurons induce immunosuppression in tumors through the actions of neuropeptide signaling (12, 14). Future research to evaluate the impact of sensory neural regulation of anti-cancer immunity may identify opportunities to improve treatment modalities that rely on a functional immune response including chemotherapy and immunotherapy. Drugs that target sensory neuropeptide signaling are already used clinically to treat migraine (gepant drugs) and nausea (pitant drugs) (101, 102), and may provide a strategy to target sensory signaling in tumors. The parasympathetic nervous system modulates anti-inflammatory pathways (103), suggesting it could be leveraged to slow cancer. However, as parasympathetic neural signaling has been implicated in tumor cell dissemination in prostate cancer (15), further research is needed. Generalized nerve blocking drugs also offer a strategy to target multiple neural subtypes within the tumor. Supporting evidence for this approach comes from a prospective clinical trial that administered lidocaine around the tumor immediately prior to surgery and found a small but significant improvement in disease-free and overall survival (104).

Opportunities for clinical translation are not limited to pharmacological strategies, with several other techniques currently being used to modulate neural signaling. While not currently used for cancer, denervation may allow targeted ablation of neurons to a specific organ. This approach is utilized to treat resistant hypertension using sympathetic denervation of the renal system via catheter (105). Additionally, implantable bioelectric devices that use electric stimulation to modulate vagus nerve signaling are currently being developed for the treatment of rheumatoid arthritis and Crohn’s Disease (106–108), suggesting that a similar approach can be used to target cancers that are regulated by the vagus nerve.

Finally, targeting neural signaling during the period of cancer treatment may have additional benefits beyond control of cancer progression. The non-selective β-blocker carvedilol is currently used to treat cardiotoxicity induced by anthracycline chemotherapy and trastuzumab (109). Carvedilol has been shown to reduce primary tumor growth and metastasis by modulating tumor cell invasion (59), raising the possibility that carvedilol could be administered at the time of conventional cancer treatment to both slow cancer progression and prevent treatment-induced cardiotoxic side-effects. Drugs that target neurotrophic signaling may be another strategy for multifaceted effects. For example, anti-NGF antibodies have been evaluated for treatment of pain (110), suggesting that they could reduce cancer-associated pain in addition to targeting the sympathetic and sensory nervous systems in the tumor microenvironment. As the NGF targeted drug tanezumab failed FDA approval for treatment of osteoarthritis (111), there may be commercial interest in repurposing this drug for treatment of cancer.

## Conclusions

Cancer is a stressful experience which elevates SNS flight-or-flight signaling in patients. In addition, cancer treatment increases tumor sensitivity to SNS signaling (23). Our understanding of how neural signaling regulates cancer treatment efficacy is expanding and will support the discovery of targeted neural interventions that may be leveraged to enhance outcomes of standard cancer treatments.
